# Consequences of Domestication on Gut Microbiome: A Comparative Study Between Wild Gaur and Domestic Mithun

**DOI:** 10.3389/fmicb.2020.00133

**Published:** 2020-02-25

**Authors:** Vandana R. Prabhu, Wasimuddin, Ranganathan Kamalakkannan, Moolamkudy Suresh Arjun, Muniyandi Nagarajan

**Affiliations:** ^1^Department of Genomic Science, School of Biological Sciences, Central University of Kerala, Kasaragod, India; ^2^Institute for Infectious Diseases, Faculty of Medicine, University of Bern, Bern, Switzerland

**Keywords:** gaur, mithun, gayal, 16S rRNA gene, microbiome, captivity, domestication

## Abstract

Although the gut microbiome benefits the host in several ways, how anthropogenic forces impact the gut microbiome of mammals is not yet completely known. Recent studies have noted reduced gut microbiome diversity in captive mammals due to changes in diet and living environment. However, no studies have been carried out to understand how the gut microbiome of wild mammals responds to domestication. We analyzed the gut microbiome of wild and captive gaur and domestic mithun (domestic form of gaur) to understand whether the gut microbiome exhibits sequential changes from wild to captivity and after domestication. Both captive and domestic populations were characterized by reduced microbial diversity and abundance as compared to their wild counterparts. Notably, two beneficial bacterial families, *Ruminococcaceae* and *Lachnospiraceae*, which are known to play vital roles in herbivores’ digestion, exhibited lower abundance in captive and domestic populations. Consequently, the predicted bacterial functional pathways especially related to metabolism and immune system showed lower abundance in captive and domestic populations compared to wild population. Therefore, we suggest that domestication can impact the gut microbiome more severely than captivity, which might lead to adverse effects on host health and fitness. However, further investigations are required across a wide range of domesticates in order to understand the general trend of microbiome shifts in domestic animals.

## Introduction

The diverse and enormously complex gut microbiome benefits animals in several ways such as by instigating immune responses, synthesizing vitamins, and carrying out metabolic functions that the host cannot perform (Bäckhed et al., 2005; Cerf-Bensussan and Gaboriau-Routhiau, 2010; LeBlanc et al., 2013; Crespo-Piazuelo et al., 2018; Nagarajan et al., 2018). Host–gut microbiome relationships are influenced by host traits such as age, sex, genotype, and extrinsic factors like diet, lifestyle, and habitat heterogeneity (Dubois et al., 2017; Wasimuddin et al., 2017). Understanding how the gut microbiome responds to these factors is important because perturbations of gut microbial communities beyond their natural range may have serious impact on the host health (Cheng et al., 2015). Recent studies in human have noted that departing from ancestral lifestyle and adapting to urban life that involves modifications of lifestyle, diet, and living environment reduces the diversity and stability of gut microbiome (Schnorr et al., 2014; Conlon and Bird, 2015; Martínez et al., 2015; Obregon-Tito et al., 2015; Rampelli et al., 2015; Valle Gottlieb et al., 2017). As observed in the case of modern human, under captive conditions, most of the animal species including mammals, birds, and amphibians experience radical shifts in their diet and living environment, sharply reduced geographic range, controlled social interactions, and increased exposure to medical interventions that contrast from their way of living in the wild (Hird, 2017; McKenzie et al., 2017; Metcalf et al., 2017; Martínez-Mota et al., 2020). In addition to these factors, domestic animals undergo substantial biobehavioral changes due to the intensive domestication process. Owing to such changes, captive and domestic animals are more likely to differ from their wild counterparts in the gut microbial diversity and composition (Hird, 2017). Furthermore, considering even altered natural state of domesticated individuals in comparison to captives, increased disturbance of microbial communities is highly probable. However, there have been no studies conducted to understand whether the gut microbiome shows sequential changes from wild to captivity and after domestication.

*Bos gaurus* commonly known as gaur or Indian bison is one of the largest extant ungulates and endemic to South and Southeastern Asia. In India, they occur in small isolated groups confined to Western Ghats, central Indian highlands, and Northeastern Himalayas (Choudhury, 2002). Although majority of gaur populations inhabit in India, it is placed in the schedule II of Indian Wild Life (Protection) Act, 1972, and considered as vulnerable species by IUCN. The domestic form of gaur is considered as a distinct species, *Bos frontalis*, and commonly known as mithun (India) and gayal (China). It is believed to have evolved from wild gaur more than 8000 years ago (Simoons, 1984; Dorji et al., 2010; Qu et al., 2012; Wang et al., 2017). However, there are different views for the origin of domestic mithun, and the recent studies have strongly supported the widely accepted view that presumes gaur as the ancestral species of domestic mithun (Mukherjee et al., 2018; Prabhu et al., 2019). The geographic range of mithun is restricted to Northeastern hilly regions of India, Myanmar, Bhutan, Bangladesh, and the Yunan province of China (Mukherjee et al., 2018). Interspecies hybridization, slaughtering, and other anthropogenic factors have led to the decline of mithun population; as a result, it is listed under the category endangered by the IUCN.

Domestic animals, particularly livestocks, are essential food resources for the world’s rapidly growing human population. Importantly, animals like cattle, sheep, goat, buffalo, mithun, etc. are able to effectively transform their forages into high-value animal products. However, animal health has been shown to greatly influence their functions by having direct effect on the productive parameters such as mortality rate, prolificacy, body weight, and milk yield and indirect effect on public health as it increases the incidence of zoonotic diseases. Hence, it is essential to understand the gut microbiome of domestic animals as distortion of microbiome has been reported to increase the incidences of diseases in human and laboratory animals. Wild and captive gaur, and domestic mithun provide an excellent biological system to study the sequential change of gut microbiome from wild to captivity and after domestication. Therefore, in order to discern the impact of captivity and domestication on gut microbiome, we examined the gut microbiome of wild and captive gaur and domestic mithun populations by sequencing the V3–V4 region of the 16S rRNA gene. We were specifically interested to investigate the following: (i) whether the wild population share similar gut microbial diversity and composition with captive and domestic populations, (ii) whether microbial taxa show sequential increase/decrease in abundance, from wild to captive and after domestication, and (iii) if anticipated shifts in the gut microbiome also reflect at their predicted functional level.

## Materials and Methods

### Sample Collection

Fecal samples of wild and captive gaur and domestic mithun were collected from different places of India. To avoid sibling effects, the samples were collected from divergent locations for each category. Fecal samples of wild gaur (*n* = 10) were collected from different places in the Western Ghats regions of Tamil Nadu and Kerala while the captive gaur samples (*n* = 10) were collected from different Zoos (Arignar Anna Zoological Park, Chennai, Tamil Nadu; Sri Chamarajendra Zoological Gardens, Mysore, Karnataka; Bannerghatta Biological Park, Bangaluru, Karnataka; Bondla Zoo, Goa; The Zoological Park, Thiruvananthapuram, Kerala). The fecal samples of domestic mithun (*n* = 10) were collected from Northeastern states of India (Nirjuli, Sagalee, and Yupia, Arunachal Pradesh, and Khuangleng, Mizoram). The fecal samples were collected in absolute ethanol within a few minutes after defecation using sterile forceps to avoid environmental contaminations and stored at −80°C until further analysis. The fecal samples were collected without having any contact with animals for which necessary permission were obtained from the respective state forest departments. Further, the fecal samples were collected with the help of respective forest officials/veterinarians in compliance with the research ethical standards of India. The study was conducted on the fecal samples and no animal was used for the purpose of this study.

### DNA Extraction and Sequencing

Genomic DNA was isolated from the fecal samples using DNeasy PowerSoil kit (Qiagen) as per the manufacturer’s instructions. The V3–V4 region of the 16S rRNA gene was amplified using the primer Pro341F (5’-CCTACGGGNBGCASCAG-3’) and Pro805R (5’-GACTACNVGGGTATCTAATCC-3’) (Nadkarni et al., 2002). The following conditions were applied for the PCR: denaturation at 98°C for 30 s, followed by 35 cycles at 95°C for 10 s, 60°C for 15 s, and at 68°C for 30 s, and final extension at 68°C for 5 min. The resulting PCR products were purified using the PureLink PCR purification kit (Thermo Fisher Scientific). The purified PCR products were proceeded with library preparation using the NEBNext Ultra DNA Lib prep kit (New England BioLabs Inc.) according to manufacturer’s instructions. The libraries were sequenced on Illumina HiSeq 2500 platform generating 2 × 250 bp paired-end reads.

### 16S rRNA Gene Sequence Data Processing

Forward and reverse reads were demultiplexed and the sequences with corresponding barcodes were merged using the software FLASH (Magoč and Salzberg, 2011). Primer sequences were removed using the software CUTADAPT (Martin, 2011). Sequences that were too long or too short were removed from the dataset using the software PRINSEQ-lite (Schmieder and Edwards, 2011). Reads were processed further using the QIIME software package (Caporaso et al., 2010) for initial quality filtering and further analysis. Sequences with quality threshold below *q* = 30 or with homopolymers or more than six ambiguous bases were discarded. The potential chimeric sequences were identified and discarded using the software USEARCH (Edgar, 2010). The Open-reference Operational Taxonomic Units (OTUs) picking approach was used to identify OTUs with a 97% similarity threshold using UCLUST (Edgar, 2010). *De novo* OTUs (i.e., reads that did not hit the Greengenes database) were also picked. The taxonomic position of OTUs was assigned using the RDP classifier (Ribosomal Database Project). The singletons OTUs and those belonging to eukaryote, archaea, mitochondria, chloroplast, and unassigned OTUs were excluded from the dataset.

### Alpha and Beta Diversity Analysis

Alpha diversity indices [number of observed species (OTUs), Chao1, and phylogenetic diversity] were calculated after rarefying the data to 58,700 sequences per sample. All further analyses were carried out in R^1^. ANOVA was performed to understand the effect of “population type (i.e., wild, captive and domestic)” on alpha diversity indices using the *lme*_4_ package (Bates et al., 2015) in R. We included population type and sampling site in the model as explanatory variables for each alpha diversity metric. The beta diversity was calculated using unweighted and weighted UniFrac metrics (Lozupone et al., 2011) methods after rarefying the data to 58,700 sequences per sample by using the *phyloseq* package (McMurdie and Holmes, 2013) in R. Permutational Multivariate Analysis of Variance (PERMANOVA) test was performed to find out the significance of the differences in the community composition with 999 permutations using *vegan* package in R. We included, as previously, population type and sampling site in the model as explanatory variables for both beta diversity metrics. Furthermore, principal coordinates analyses (PCoA) were performed based on UniFrac metrics to understand the pattern of separation between different populations. To deduce the effect of population type on inter-individual variability, Wilcoxon rank-sum tests were performed.

### Identification of Major Gut Bacterial Phyla and OTUs

In order to understand the bacterial phyla that were specifically influenced by population type, we performed ANOVA on relative abundance of the predominant phyla including population type and sampling site in the models as explanatory variables as previously described. To identify the OTUs accountable for differences among populations, we employed a negative binomial model-based approach available in the *edgeR* package (Robinson et al., 2010) in R after removing OTUs that were present in less than three samples for each population. Exact tests (Exact binomial test generalized for over dispersed counts) were performed and only OTUs that remained significant (*p* < 0.01) after the Benjamini–Hochberg correction were reported.

### Microbiome Functional Predictions

PICRUSt (Langille et al., 2013) was used to predict the functional differences of the gut microbiome. The metagenome prediction was performed using KEGG Orthology (KOs) classification after removing all *de novo* OTUs and normalization for copy number variation. To ensure the accuracy of the prediction, weighted Nearest Sequenced Taxon Index (NSTI) scores were estimated. The average NSTI values for wild gaur, captive gaur, and domestic mithun were adequately low (mean NSTI = 0.20 ± 0.02 s.d.) to enable accurate prediction of metagenomes. To investigate the effect of population type on the KEGG composition, we calculated “*Euclidean*” and “*Jaccard*” distances after rarefying the data using the package *phyloseq* (McMurdie and Holmes, 2013) in R. PERMANOVA was performed to check the significance of the differences in the KEGG composition using 999 permutations. Population type and sampling site were included in the model as explanatory variables to explain differences in the distance metrics. PCoA plots were drawn to demonstrate the differences between populations. We classified the KOs into major functional pathways by applying the KEGG classification at the hierarchy level 2. We carried out Exact tests implemented in the *edgeR* package (Robinson et al., 2010) in R to find out the pathways, which show differential abundance based on population type, and only pathways that remained significant (*p* ≤ 0.05) after Benjamini–Hochberg correction were reported.

## Results

### Microbiome Composition and Diversity

**FIGURE 1:**
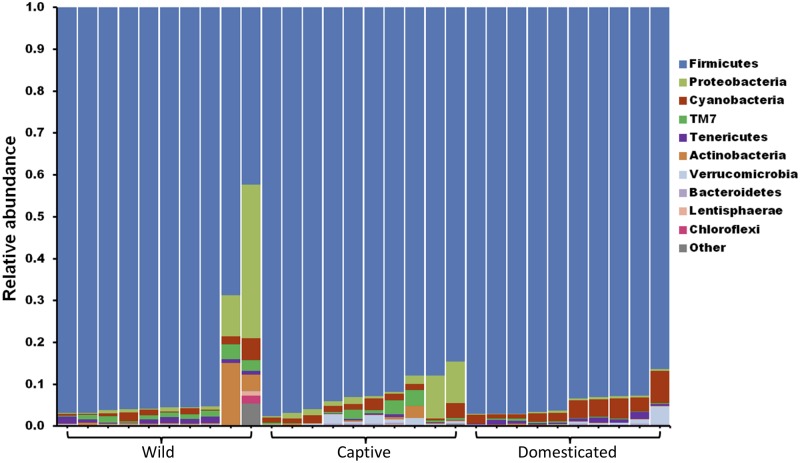
The gut microbiome composition of gaur and mithun at phylum level. Each bar represents the relative abundance of different phyla in individual samples. Each color represents one of the 10 most abundant phyla in all samples. All other bacteria are grouped as others.

The gut microbiome of wild and captive gaur and domestic mithun was characterized by sequencing the V3–V4 hypervariable region of 16S rRNA gene. In total, 4,520,177 high-quality reads with an average of 150,672 reads per sample were used for analysis after quality filtering. Microbiome of all the three populations was constituted mainly by the following 10 bacterial phyla, Actinobacteria, Bacteroidets, Chloroflexi, Cyanobacteria, Firmicutes, Lentisphaerae, Proteobacteria, TM7, Tenericutes, and Verrucomicrobia (Figure 1). Among these, Firmicutes (91.5%) was the predominant phyla followed by Proteobacteria (2.8%), Cyanobacteria (2.1%), TM7 (0.9%), and Actinobacteria (0.9%).

**FIGURE 2:**
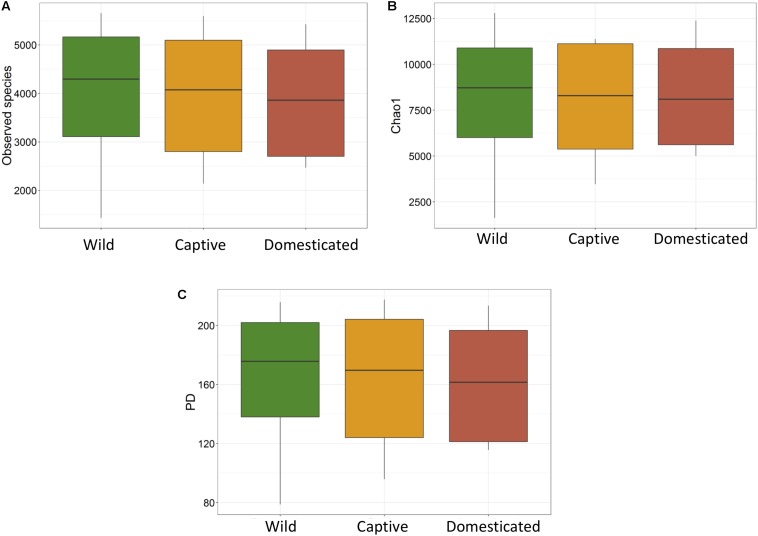
Microbial alpha diversity of gaur and mithun. **(A)** Number of observed species (OTUs), **(B)** Chao1, **(C)** phylogenetic diversity.

**FIGURE 3:**
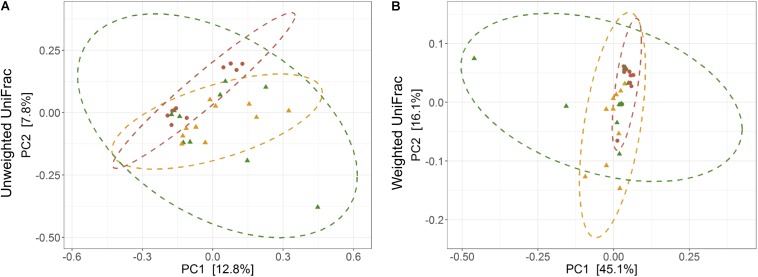
Effect of domestication on bacterial community composition. Principal coordinates analysis plots show **(A)** unweighted and **(B)** weighted UniFrac distances in wild, captive, and domestic populations (PERMANOVA: unweighted *R*^2^ = 0.111, *p* = 0.001; weighted *R*^2^ = 0.144, *p* = 0.013). Dots and surrounding dashed ellipses (95% confidence level) represent the gut bacterial communities of wild (green), captive (yellow), and domestic (red) populations.

**FIGURE 4:**
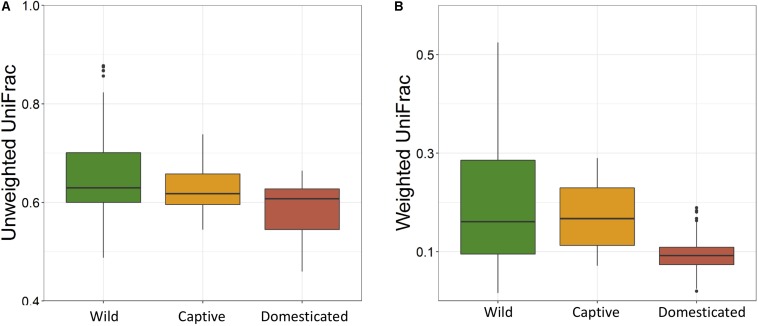
Effect of domestication on the inter-individual beta diversity of gaur. The box plots show the beta-diversity distances between individuals of wild (green), captive (yellow), and domestic (red) populations for both **(A)** unweighted (Kruskal–Wallis test: *p* < 0.001) and **(B)** weighted (Kruskal–Wallis test: *p* < 0.001) UniFrac tests.

The average observed species count, microbial richness (Chao1 index), and diversity (Shannon index) were found to be 3948, 8218, and 8.09, respectively. Further, ANOVA models explained that there is no significant variation (*p* > 0.05) in the alpha diversity estimates between wild, captive, and domestic populations (Figure 2). However, microbial community composition was significantly influenced by population type as revealed by PERMANOVA models using both unweighted (*R*^2^ = 0.111, *p* = 0.001) and weighted (*R*^2^ = 0.144, *p* = 0.013) UniFrac distances. Sampling site showed significant (*R*^2^ = 0.464, *p* = 0.023) effect on unweighted UniFrac distance but failed to show significant effect on weighted UniFrac distance (*R*^2^ = 0.479, *p* = 0.223). The PCoA explained 20.6 and 61.2% variation between populations for unweighted and weighted UniFrac distances, respectively (Figure 3). The inter-individual beta diversity varied significantly (*p* < 0.001) in all the three populations. Among the three populations, domestic population showed lowest beta diversity index, which indicates high level of similarity in the microbial composition of domestic population (Figure 4).

### Relative Abundance of Major Phyla and OTUs

**FIGURE 5:**
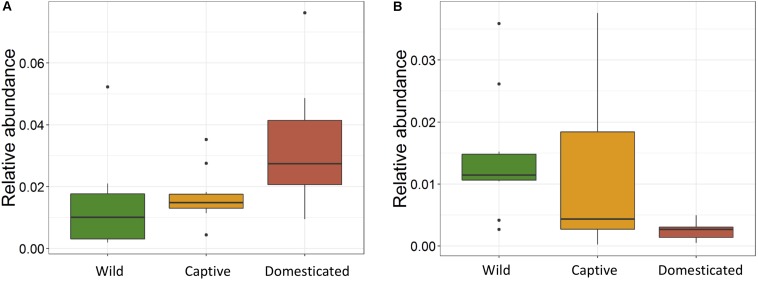
Effect of captivity and domestication on the relative abundance of major bacterial phyla. Box plots indicate the effect of domestication on the relative abundance of two major phyla. **(A)** Cyanobacteria (*p* = 0.001) and **(B)** TM7 (*p* = 0.03) in wild (green), captive (yellow), and domestic (red) populations.

**FIGURE 6:**
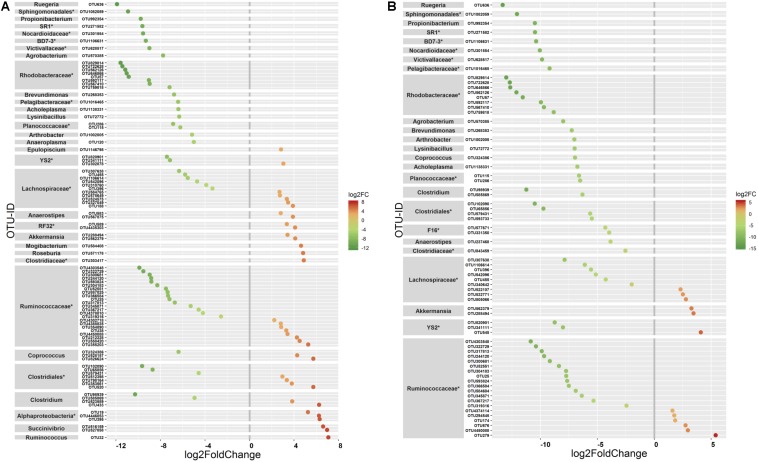
Differential abundance of OTUs between gaur and mithun. OTUs that differed in their mean abundance with respect to population type were filtered by negative binomial Exact test. **(A)** Wild and captive gaur, **(B)** wild gaur and domestic mithun. OTUs were arranged according to increasing values of log-fold change. The *X* axis shows the log_2_fold (logFC) decrease (green) and increase (red) of the OTUs based on the population type. The highest possible taxonomic rank is assigned for each OTU. *Indicates unclassified OTUs at genus level.

The ANOVA tests showed remarkable differences between populations in the proportion of Cyanobacteria (*p* = 0.001) and TM7 (*p* = 0.03) phyla. The relative abundance of Cyanobacteria (Figure 5A) increased from wild to domestic population, whereas TM7 showed the opposite trend (Figure 5B). Similarly, several OTUs (108) showed substantial differences in the mean abundance between wild, captive, and domestic populations. Between the wild and captive populations, 91 OTUs showed differential abundance, of which 53 OTUs (58%) revealed decrease in abundance and 38 OTUs (42%) revealed increase in abundance in the captive population (Supplementary Table S1). The OTUs that were underrepresented mainly belonged to the families *Ruminococcaceae*, *Lachnospiraceae*, and *Rhodobacteraceae*, while the OTUs belonging to the genera *Anaerostipes*, *Succinivibrio*, and *Akkermansia* showed increase in abundance (Figure 6A). Similarly, 68 OTUs showed differential abundance between wild and domestic populations. Among the 68 OTUs, 56 (82%) showed decrease in abundance whereas only 12 (18%) showed increase in abundance in domestic population (Supplementary Table S2). The decreased abundance was noticed mainly for the OTUs related to the *Ruminococaceae*, *Rhodobacteraceae*, and *Lachnospiraceae* family (Figure 6B). Among the 108 differentially abundant OTUs, 44 OTUs sequentially declined in abundance from wild to captive and further in domestic population. Majority of these OTUs belonged to the families *Ruminococaceae* (*n* = 12), *Rhodobacteraceae* (*n* = 8), and *Lachnospiraceae* (*n* = 4).

### Predicted Functional Pathways

**FIGURE 7:**
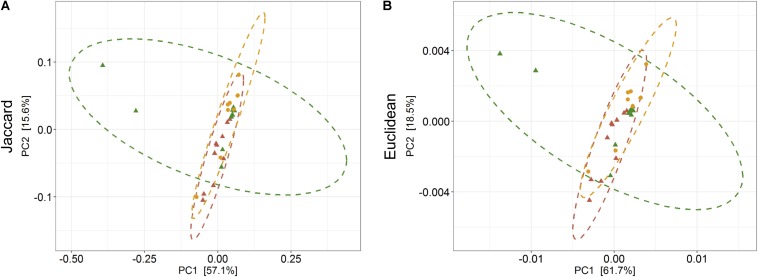
Effect of captivity and domestication on the predicted KEEG orthologs (KOs) of gaur and domestic mithun. Principal coordinates analysis plots show **(A)** Jaccard and **(B)** Euclidean distances based on the predicted KOs in wild, captive, and domestic populations (PERMANOVA: Jaccard *R*^2^ = 0.157, *p* = 0.033, Euclidean *R*^2^ = 0.144, *p* = 0.027). Dots and dashed ellipses (95% confidence level) reflect the predicted KOs of wild (green), captive (yellow), and domestic (red) populations.

**FIGURE 8:**
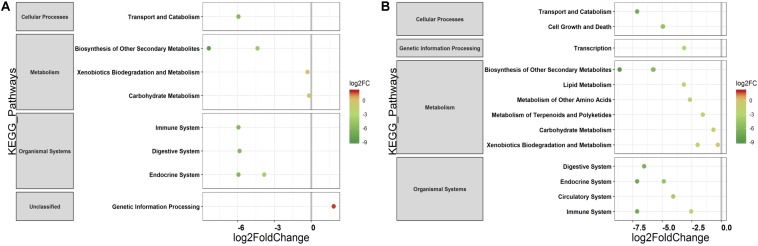
Differential abundance of predicted major functional pathways in gaur and domestic mithun. Differences in the mean abundance of major functional pathways (identified by PICRUSt prediction by using KEGG classification) identified by Exact tests (*p* < 0.05) that differ between **(A)** wild and captive gaur and **(B)** wild gaur and domestic mithun. The *X* axis shows log_2_fold (logFC) decrease (green) and increase (red) in relative abundance. Functional pathways are arranged according to increasing values of logFC.

The PERMANOVA was performed to examine whether differences in the predicted KEGG Orthologs (KOs) could be explained by population type or sampling site. Both Jaccard and Euclidean distances, based on predicted KOs, showed significant differences between populations (Jaccard *R*^2^ = 0.157, *p* = 0.033; Euclidean *R*^2^ = 0.144, *p* = 0.027) but not on sampling sites (Jaccard *R*^2^ = 0.492, *p* = 0.223; Euclidean *R*^2^ = 0.464, *p* = 0.284). The PCoA explained 72.7 and 80.2% variance for Jaccard and Euclidean distances, respectively (Figure 7). KEGG analysis identified eight functional pathways that showed differential abundance between wild and captive populations (Exact test, *p* < 0.05). All the identified pathways (“transport and catabolism,” “digestive system,” “biosynthesis of other secondary metabolites,” “endocrine system,” “xenobiotics biodegradation metabolism,” and “immune system”) showed decrease in abundance in captive population except the pathway related to “genetic information processing” (Figure 8A), whereas 13 pathways exhibited differential abundance between wild and domestic populations (exact test, *p* < 0.05). Surprisingly, all the pathways corresponding to “cell growth and death,” “endocrine system,” “circulatory system,” “transcription,” “lipid metabolism,” “carbohydrate metabolism,” “xenobiotics biodegradation and metabolism,” “metabolism of terpenoids and polyketides,” and “immune system” showed decrease in abundance in domestic population (Figure 8B).

## Discussion

A good number of studies have previously examined the gut microbial diversity between wild and captive populations. In most of these studies, the microbial diversity significantly reduced in the captive animals compared to their wild counterparts, suggesting diet and environment as probable factors for such reduction. Domestication of a species could be considered as a successive step after initially keeping the animals in captivity, which might further influence the gut microbiome. However, so far, no study has accounted all three scenarios simultaneously, i.e., compared the gut microbiome variations between wild, captive, and domestic populations. This is the first study to characterize the composition and structure of gut microbiome of wild, captive, and domestic populations and to provide important implications for the conservation and management of wild and domestic species. The gut microbiome of gaur and mithun contained Firmicutes as the dominant phylum. The occurrence of phylum Firmicutes in higher abundance was in accordance with previous studies on other ruminants such as cattle, goat, sheep, wood bison, and alpaca (De Menezes et al., 2011; Weese et al., 2014; O’Donnell et al., 2017). Firmicutes is the widely reported phylum in the mammalian gut and is known to have a significant role in host metabolism and digestion (Ley et al., 2008). Members of Firmicutes are particularly capable of degrading a wide range of polysaccharides (Cockburn and Koropatkin, 2016); hence, the higher abundance of Firmicutes can be correlated with the food habits of gaur and mithun. Similarly, the presence of other bacterial phyla was also in accordance with previous studies (Weese et al., 2014; O’Donnell et al., 2017).

There was no significant difference in the alpha diversity measures between wild, captive, and domestic populations. Numerous, previous studies have also observed no difference in the alpha diversity between wild and captive populations of rhinoceros, musk deer, bovid, giraffes, aardvarks, and anteaters (Li et al., 2017; McKenzie et al., 2017; Gibson et al., 2019). In contrast, beta diversity significantly varied between populations, suggesting that distinct group of microbes inhabits the gut of wild, captive, and domestic populations. In particular, a gradual decline was observed in the microbial diversity from wild to domestic population. It indicates that the gut microbial diversity is sequentially lost in gaur during domestication, beginning with reduction of microbial diversity to some extent when the animal was moved from wild to captivity (the first step taken toward domesticating an animal) and then losing a much greater portion of the microbiome at the later stage of domestication process. However, the effect of location on gut microbiome was weak and limited to unweighted UniFrac distance only. Also, location did not show any effect on predicted microbial functions, suggesting that overall effect of location is weaker compared to that of population type. It is to be expected that beta diversity will be higher in wild population as they naturally feed on a vast variety of plant species including grasses, herbs, shrubs, and trees in large quantities to meet their daily energy requirements, and the samples were also collected from divergent locations. The relatively low beta diversity of captive population, although they were sampled from more diverse locations than wild population, suggested that not locations but captivity-induced factors such as similar diet, limited geographical space, and contact with conspecific and similar artificial environment in zoos probably constrain the gut microbiome, making it more similar between individuals (Clayton et al., 2016; Billiet et al., 2017; Rosenfeld, 2017; Hale et al., 2018). However, the low beta diversity of domestic population cannot be solely attributed to the above mentioned factors because generally mithun are allowed to roam freely in the forests, where they graze and browse upon a vast variety of plant species (Mondal et al., 2014). Also, the diet of domestic mithun and wild gaur is similar, which includes plants mainly from *Poaceae* and *Fabaceae* families (Nayak and Patra, 2015; Haleem and Ilyas, 2018; Jamir and Khare, 2018), which suggests that diet alone might not be the causative factor for the low beta diversity of domestic mithun.

The relative abundance of Cyanobacteria and TM7 remarkably differed between three populations. Cyanobacteria showed increasing trend in captive and domestic populations compared to wild population. Cyanobacteria are aerobic bacteria widely observed in aqueous and soil environments and are capable of fermenting a range of sugars in anoxic conditions (Nandi and Sengupta, 1998; Williams et al., 2004; Cruz-Martínez et al., 2009). As captive gaur and domestic mithun live in the vicinity of human settlements, starches probably might have become a regular part of their diet. The increase of Cyanobacteria therefore could be an indication that captive and domestic populations are acquiring gradual adaptation in response to the increasing starch content in their diet as reported previously in dog (Axelsson et al., 2013). In contrast, the relative abundance of TM7 phyla decreased in captive and domestic populations. The decreasing trend of TM7 phyla has also been observed in captive Javan slow loris individuals fed with normal diet compared to individuals fed with improved diet (Cabana et al., 2019). TM7 bacteria are found in diverse habitats like soil, freshwater, human oral cavity, gut of several animals, etc. (Marcy et al., 2007; Kuehbacher et al., 2008; Ferrari et al., 2014); however, the functional attributes of TM7 phylum remain largely unknown. Some members of TM7 have been suggested to presumably play a role in the degradation of polyphenols in the gut of woodrat (Kohl et al., 2011). Therefore, the reduction of TM7 may affect the digestion efficiency of captive and domestic populations as polyphenols; in particular, tannins at higher concentrations are reported to reduce the nutrient absorption in ruminants (Frutos et al., 2004).

At the lower taxonomic ranks, many OTUs significantly differed in relative abundance between wild, captive, and domestic populations, and most of these differences observed were characterized by decrease in the abundance of OTUs. The majority of bacterial OTUs that showed decreasing trend in captivity and after domestication belonged to the families *Ruminococcaceae*, *Rhodobacteraceae*, and *Lachnospiraceae*. The members of *Ruminococcaceae* and *Lachnospiraceae* families are known to have high number of glycoside hydrolase genes that enable them to break down the complex plant components such as cellulose, hemicellulose, and other polysaccharides (Biddle et al., 2013). In herbivorous animal, the bacterial breakdown of complex plant materials has been reported to account for more than 50% of their energy production (Flint et al., 2008). Hence, the reduction of commensal *Ruminococcaceae* and *Lachnospiraceae* might seriously affect the dietary energy requirements of captive and domestic populations. Other than impeding the efficiency of host digestion, these bacterial families are also associated with protection against enteric infections (Wlodarska et al., 2015). *Rhodobacteraceae* are reported to be involved in water purification, which removes harmful substances from water (Nupur et al., 2013) and known to be major producers of vitamin B_12_ in marine ecosystems (Sañudo-Wilhelmy et al., 2014). Some members of *Rhodobacteraceae* are able to produce tropodithietic acid, which inhibits the growth of pathogens (Beyersmann et al., 2017). However, their functional attributes in the rumen is not fully known.

Domestication is a process in which a subset of wild animals are selected artificially for their desired phenotype over a period of time for human needs. In the course of domestication, wild animals undergo prolonged period of stress and behavioral changes. Recent studies have shown that many factors associated with domestication can either directly or indirectly influence the gut microbiome in domestic animals (Yuan et al., 2015; Cunningham et al., 2018; Karl et al., 2018; Griffiths et al., 2019; Li et al., 2019). Our study showed significant differences in the gut microbiome between wild and domestic populations, and these differences cannot be solely attributed to the diet and environment as reported in previous studies, because the diet of domestic mithun and wild gaur is similar and the effect of location was not significant in our analyses. Therefore we suggest that the microbiome differences observed between wild and domestic populations can be attributed to the domestication associated factors such as artificial selection, inbreeding, phenotype, genotype, physiological changes, stress, etc. Among these factors, inbreeding is an unavoidable consequence in domestication due to the intense selection process in which only a few superior males are allowed to breed with females. Recent studies have shown the influence of inbreeding on gut microbiome composition in house mice and gopher tortoises (Kreisinger et al., 2014; Yuan et al., 2015). The phylum Firmicutes showed decreased abundance in inbred individuals in both the studies. Similarly, in our study, several OTUs particularly belonging to the phylum Firmicutes also decreased in abundance in domestic mithun as compared to wild population. Most of the Firmicutes OTUs were represented by the families *Lachnospiraceae* and *Ruminococcaceae*, which are known to be beneficial to the host in several ways particularly associated with host metabolism and defense mechanism (Biddle et al., 2013; Barelli et al., 2015). Therefore, we assume that, as a consequence, the pathways associated with digestive and immune systems were predicted to be low in domestic mithun. Reduction of such functionally relevant microbes that aid in host digestion and defense mechanism likely points at the adverse impact of artificial selection/inbreeding on the gut microbiome. However, carefully planned experimental laboratory animal crosses based on chosen phenotypic traits and keeping other variables in control can better reveal the role of artificial selection on the gut microbiome. Given the importance of gut microbiome in facilitating immune functions, it is possible that domestication might perturb the host immune response and cause pathogenesis, thereby having a direct effect on the production performances of domestic animals. Thus, further investigations are required in this area to dissect out the influence of each factor associated with domestication on gut microbiome.

## Conclusion

Our results showed significant variations in the gut microbiome between wild, captive, and domestic populations. These variations, to a great extent, were characterized by low bacterial diversity and significant loss of several bacterial OTUs predominantly belonging to commensal bacteria. Although such variations are generally explained by radical shifts in the diet, our study shows that microbiome variations in the domestic population could also be attributed to domestication process. If domestication exerts such an impact on the gut microbiome of domestic mithun, even though they are allowed to roam freely in the forest to compensate their limited diet, it might have serious impact on gut microbiome of the animals that are raised under strict human constructed environments. Our findings therefore imply that domestication might affect the health and fitness of animals by altering the gut microbiome. However, it is necessary to study the gut microbiome variations associated with domestication across a wide range of domesticates in order to understand the general trend of microbiome shifts in domestic species. Studies of such kind may indeed have broader implications in the health management and conservation of wild and domestic animals.

## Data Availability Statement

The raw sequence reads can be found at Sequence Read Archive (SRA) under BioProject ID: PRJNA595722.

## Author Contributions

MN conceived the study. VP performed the experiments and wrote the manuscript. Wasimuddin performed the analyses. VP, RK, and MA contributed to sample collection. Wasimuddin and MN revised the manuscript. All authors read and approved the final manuscript.

## Conflict of Interest

The authors declare that the research was conducted in the absence of any commercial or financial relationships that could be construed as a potential conflict of interest.

## Footnotes

1 https://www.r-project.org
